# A Case of Opiate-Induced Toxic Leukoencephalopathy in a Middle-Aged Woman

**DOI:** 10.7759/cureus.39113

**Published:** 2023-05-16

**Authors:** Dipraj Limbu, Jeyanthy Rajkanna, Mayada Abdelrahman, John Kamara

**Affiliations:** 1 Acute Medicine, Peterborough City Hospital, Peterborough, GBR; 2 Diabetes and Endocrinology, Peterborough City Hospital, Peterborough, GBR; 3 Internal Medicine, Peterborough City Hospital, Peterborough, GBR; 4 Cardiology, Peterborough City Hospital, Peterborough, GBR

**Keywords:** spongiform, white matter, cerebral, opioids, leukoencephalopathy, toxic

## Abstract

Toxic leukoencephalopathy is a disorder characterized by the alteration of myelin in white matter tracts secondary to exposure to neurotoxic substances. Here we describe a case of a middle-aged woman who presented to the emergency department with a history of bizarre behaviour, speech abnormalities and generalised muscle stiffness caused due to recent opioid overdose. Further investigations and extensive neurological tests, including magnetic resonance imaging (MRI) scan of the brain, demonstrated features consistent with toxic leukoencephalopathy (TLE). The patient was managed conservatively with the care of a multidisciplinary team involving a dietician, physiotherapist and speech and language therapist. She showed gradual and slow but significant recovery following a period of neurorehabilitation. The clinical presentation of TLE varies but MRI typically shows diffuse bi-lateral white matter lesions. History of neurotoxin exposure, presenting clinical signs and symptoms and radiological findings are significant in making the diagnosis. Early recognition is crucial and can help optimize patient’s recovery and prevent severe complications.

## Introduction

Toxic leukoencephalopathy (TLE) describes a spectrum of clinical and histopathological features associated with structural changes to cerebral white matter injured by a leukotoxic agent. Such substances include drugs of abuse, antineoplastic drugs, immunosuppressive drugs, antimicrobial agents and environmental toxins [1, 2]. Although the exact mechanism of toxicity remains speculative, patterns of injury have been described. Histopathology demonstrates white matter vacuolization and spongiform defects as hallmarks of TLE [1-3]. The detection of early and subtle toxin effects has been facilitated by magnetic resonance imaging, which offers a better resolution of white matter than other neuroimaging methods and continues to offer important insights into its nature. Its clinical presentation may vary from inattention and personality changes to coma and death [1]. We describe a patient who was found to have opiate-induced TLE.

## Case presentation

Medical history and demographics

A 51-year-old lady was brought into the emergency department by ambulance with an altered mental status. She was found by neighbours disoriented. She had a medical history of emotionally unstable personality disorder superseding a diagnosis of schizophrenia, post-traumatic stress disorder, recreational drug use and alcohol excess, and a history of drug overdose. She lived alone in a warden-controlled flat. Her regular medications included: codeine phosphate 15 milligram four times a day, temazepam 20 milligram once a day, topiramate 75 milligram twice a day, omeprazole 20 milligram once a day, venlafaxine 225 milligram once a day, pregabalin 300 milligram twice a day and cetirizine 10 milligram once a day.

Examination revealed pinpoint pupils, a low Glasgow coma scale (GCS) of 9/15 (Eye opening: 2, Verbal response: 3, Motor response: 4), a raised temperature of 38.4-degree Celsius, oxygen saturation of 85% on air and tachycardia. Further, respiratory, cardiovascular and abdominal examinations were unremarkable. Her GCS significantly improved to 14/15 after she was given 400 micrograms of naloxone.

She was admitted to the medical ward for the treatment of presumed urinary tract infection (urine dip positive for leucocytes and nitrites but urine culture was negative) and acute kidney injury secondary to rhabdomyolysis (creatine phosphokinase 3462). The cause of her altered mental status was not fully established, but it was felt to be likely secondary to infection and drug overdose. During her brief admission, her delirium resolved fully. On discharge, all her regular medications from admission were continued except codeine phosphate.

Fourteen days after discharge, she re-presented with a dramatic decline in cognition and erratic behaviour. Initial GCS was 14/15 (Eye opening: 4, Verbal response: 4, Motor response: 6). During this second admission she confided that prior to her previous admission, she took excess of her medication which was given to her for pain but declined to mention anything in detail. In the following week, her symptoms progressed to mutism with some resistance to movement of proximal upper limbs along with frontal lobe symptoms which included dystonia, gait abnormality, disorientation, confabulation, disinhibition and preservation.

Investigations

**Figure 1:**
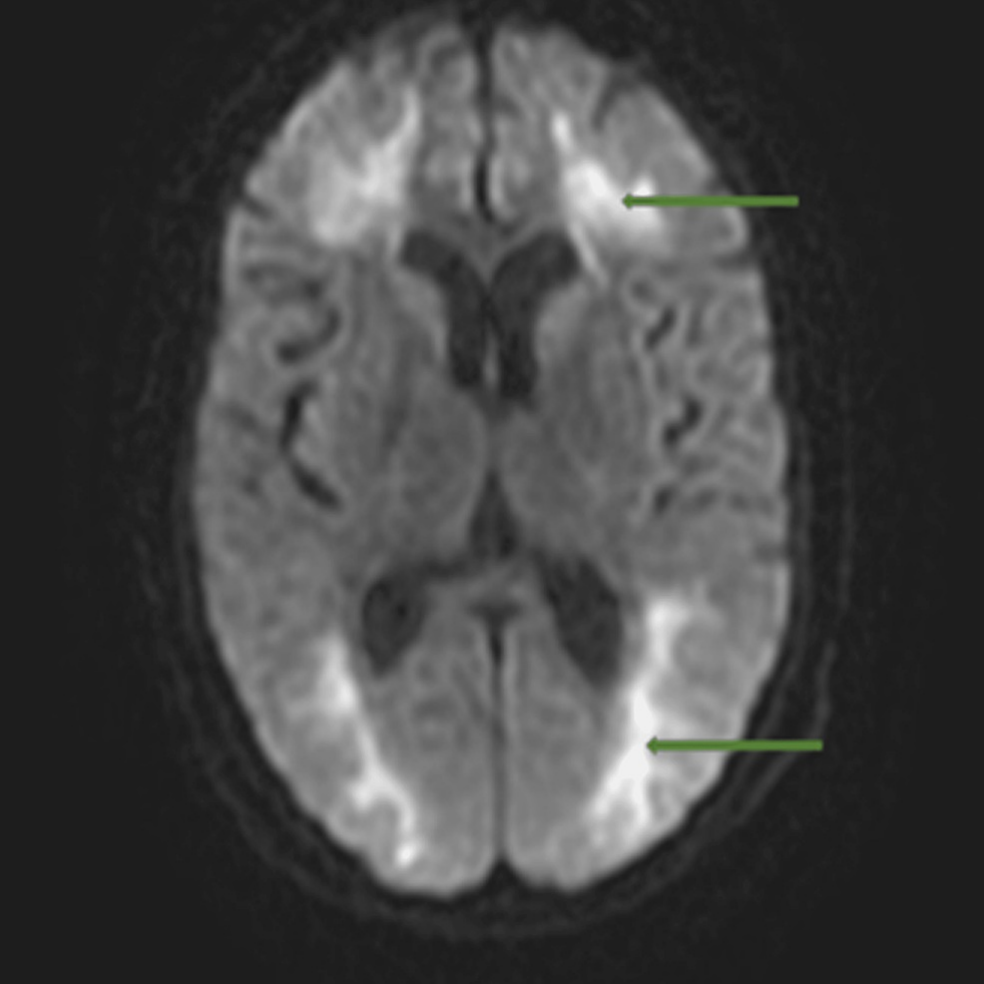
Magnetic resonance imaging (MRI) AX DWI showing diffuse bilateral cerebral white matter changes (green arrows) involving the frontal, parietal, occipital, and temporal lobes.

**Figure 2:**
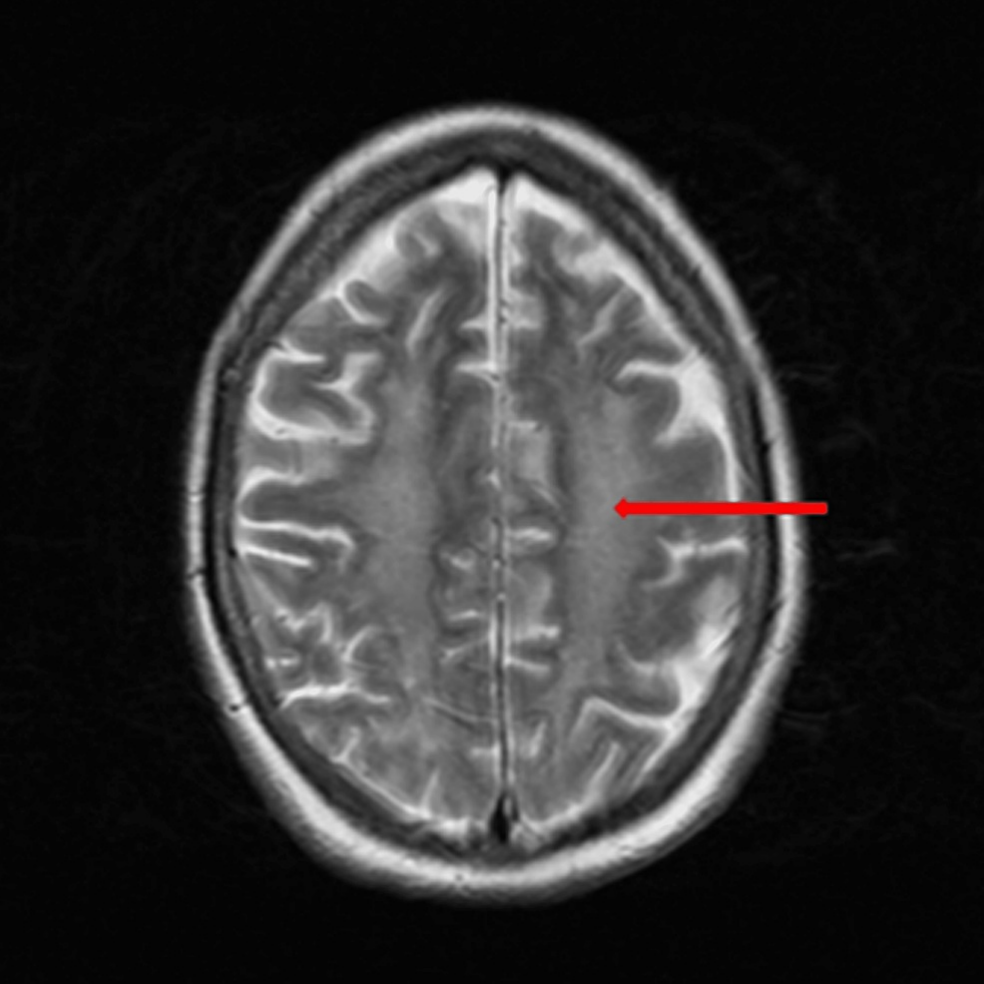
Magnetic resonance imaging (MRI) AX T2 showing diffuse bilateral cerebral white matter changes (red arrow) involving the frontal, parietal, occipital, and temporal lobes.

An initial plain CT head and with contrast revealed mild background global brain evolutionary changes but no acute intracranial pathology was detected. Cerebrospinal fluid (CSF) analysis demonstrated slightly raised protein and glucose with no evidence of central nervous system (CNS) infection. Further neuroimaging with brain MRI showed extensive leukoencephalopathy consistent with a toxic cause (Figures 1, 2), and given her clinical presentation of pinpoint pupils that responded well to the administration of naloxone and history of previous drug abuse, opioid overdose was the most favourable diagnosis. She was also investigated extensively for other causes of leukoencephalopathy (Table 1).

**Table 1:** Results of investigations done to rule out causes of leukoencephalopathy CSF: Cerebrospinal fluid; TSH: Thyroid stimulating hormone

Investigation	Result	Normal range
CSF Microscopy	No organism seen	
CSF cultures	Clear colour fluid, no growth after extended incubation	
Blood culture	No growth	
Anti-glutamic acid decarboxylase IgG antibodies	<5.0 IU/mL	<10 IU/mL
Paraneoplastic antibody profile	Negative	
Antibody to HIV I & II	Not detected	
Treponema pallidum Ab	Negative	
White cell enzymes	Normal activity	
Plasma Amino Acids	No evidence for an amino acidopathy	
Autoimmune screen	Negative	
TSH	1.27 mU/L	0.30 – 4.20 mU/L
Alpha fetoprotein	4 ng/ml	<7 ng/ml
Anti-TPO	19 IU/ml	<34 IU/ml
Ca125	22 U/ML	<35 U/ML
Folate	4.3	>3.9
B12	274	197 – 771
CT chest abdomen and pelvis	No evidence of malignancy seen	
Ultrasound abdomen, kidneys, ureters and bladder	Normal pancreas, liver, spleen and kidneys	

Treatment

Our patient was managed conservatively with nutritional supplements based on the recommendations made by the speech and language therapist and dietetics teams. She received baclofen for increased muscle tone and was supported by physiotherapists and occupational therapists with muscle-strengthening exercises. She was bedbound for a considerable period and continued to exhibit neuropsychiatric sequelae of her brain injury with clear frontal lobe symptoms requiring psychiatric support. She was perseverating in her speech, somewhat disinhibited and confabulating. She was also disoriented in time, place and person.

Outcome and follow-up

Two months after her admission, her verbal communication improved, mobility improved from being bedbound to walking with a frame independently. She was referred to a specialist neuro-rehabilitation centre on discharge for continuation of care. She is doing well, living independently in her own house.

## Discussion

Toxic leukoencephalopathy (TLE) was first reported in 1982 and has been found to be associated with exposure to a range of toxins from therapeutic agents to illicit substances; these agents mainly affect white matter tracts, causing clinical symptoms ranging from disorientation to dementia and even death [4]. The prevalence of TLE is found to be very low because cases are often underreported, mainly when associated with drug abuse and overdose. While the exact mechanism and pathogenesis of TLE remain unclear, the most common impression is that it involves a hypoxic-ischemic injury to white matter causing spongiform degeneration [4]. Magnetic resonance imaging is the best investigation modality used for the diagnosis of TLE and it typically shows bi-hemispheric white matter changes (Figures 1, 2) [5]. Clinical outcomes may vary from full recovery in some, over a period of three months to one year, to death in others [6, 7].

One common factor associated with abusable substances is their high lipid solubility, allowing easy access to the brain [8]. Our patient was admitted with a suspected opioid overdose and nearly two weeks after her initial recovery she was found to have symptoms and neuroradiological abnormalities consistent with TLE. The analgesic effect of opiates derives from the activation of kappa and delta receptors; these combined effects along with euphoria and drowsiness occur after the activation of Mu receptor in the nucleus accumbens and it is these effects that are commonly sought by drug abusers [9].

In any patient presenting with new onset neurobehavioral changes after exposure to toxins like heroin, cocaine, including other illicit stimulants and abusable drugs, TLE should be taken into consideration as one of the differential diagnoses [4]. The other clinical conditions which might present like TLE and need distinguishing from include posterior reversible encephalopathy syndrome, hypoxic-ischemic encephalopathy, meningitis, encephalitis, stroke and other metabolic syndromes. Recognizing this rare clinical condition is important because some study findings suggest potential clinical and radiological reversibility to a certain extent [10]; early recognition can help clinicians to facilitate treatment along with supportive therapy and removal of the offending substance in the early stages. Clinicians need to be aware of the possibility of delayed-onset presentation of TLE cases, usually with psychiatric symptoms, and key part of a multidisciplinary team in enabling recovery [4].

## Conclusions

This case highlights the clinical presentation and evaluation of a patient with toxic leukoencephalopathy. MRI imaging is the diagnostic investigation of choice. There may be a latent period between toxin exposure and the development of symptoms and signs of toxic leukoencephalopathy. Anticipation and early recognition are significant in the prevention of potentially severe complications of TLE and in facilitating effective recovery in patients.
